# Age-related retention of fiber cell nuclei and nuclear fragments in the lens cortices of multiple species

**Published:** 2011-10-15

**Authors:** William Pendergrass, Galynn Zitnik, Silvan R. Urfer, Norman Wolf

**Affiliations:** Department of Pathology, University of Washington, Seattle, WA

## Abstract

**Purpose:**

To determine the differences between species in the retention of lens fiber cell nuclei and nuclear fragments in the aging lens cortex and the relationship of nuclear retention to lens opacity. For this purpose old human, monkey, dog, and rat lenses were compared to those of three strains of mouse. We also investigated possible mechanisms leading to nuclear retention.

**Methods:**

Fixed specimens of the species referred to above were obtained from immediate on site sacrifice of mice and rats, or from recently fixed lenses of other species, dogs, monkeys, and humans, obtained from collaborators. The retention of undegraded nuclei and nuclear fragments was graded 1–4 from histologic observation. All species lenses were examined microscopically in fixed sections stained with hematoxylin and eosin (H&E) or 4',6-diamidino-2-phenylindole (DAPI). Slit lamp observations were made only on the mice and rats before sacrifice and lens fixation. Values of 0 to 4 (clear lens to cataract) were given to degree of opacity. MRNA content in young versus old C57BL/6 mouse lenses was determined by quantitative PCR (qPCR) for DNase II-like acid DNase (*DLAD*) and other proteins. DLAD protein was determined by immunofluorescence of fixed eye sections.

**Results:**

In old C57BL/6 and DBA mice and, to a lesser degree, in old CBA mice and old Brown Norway (BN) rats lenses were seen to contain a greatly expanded pool of unresolved whole nuclei or fragments of nuclei in differentiating lens fiber cells. This generally correlated with increased slit lamp opacities in these mice. Most old dog lenses also had an increase in retained cortical nuclei, as did a few old humans. However, a second rat strain, BNF1, in which opacity was quite high had no increase in retained nuclei with age nor did any of the old monkeys, indicating that retained nuclei could not be a cause of opacity in these animals. The nuclei and nuclear fragments were located at all levels in the outer cortex extending inward from the lens equator and were observable by the DAPI. These nuclei and nuclear fragments were seen from 12 months onward in all C57BL/6 and DBA/2 mice and to a lesser degree in the CBA, increasing in number and in space occupancy with increasing age. Preliminary results suggest that retention of nuclei in the C57BL/6 mouse is correlated with an age-related loss of DLAD from old lenses.

**Conclusions:**

A very marked apparently light refractive condition caused by retained cortical nuclei and nuclear fragments is present in the lens cortices, increasing with age in the three strains of mice examined and in one of two strains of rats (BN). This condition was also seen in some old dogs and a few old humans. It may be caused by an age-related loss of DLAD, which is essential for nuclear DNA degradation in the lens. However, this condition does not develop in old BNF1 rats, or old monkeys and is only seen sporadically in humans. Thus, it can not be a universal cause for age related lens opacity or cataract presence, although it develops concurrently with opacity in mice. This phenomenon should be considered when using the old mouse as a model for human age-related cataract.

## Introduction

The anterior central region of the lens is covered with nucleated amitotic lens epithelial cells (LEC). Lateral to this lies a ring of mitotic LEC, which subsequently migrate to the equator at the lens surface, elongate, and enter into the outer cortex where they become lens fiber cells and continue a program of differentiation into secondary lens fibers (lens fibers accreted after adulthood). This differentiation process includes degradation and removal of lens organelles, and expression of lens crystallins and other lens specific proteins. As lens fiber cell accumulation progresses the nuclei and interior organelles are lost and serial layers of interiorized lens fibers are laid down, burying the more secondary lens fibers deeper in the cortex. This process maintains the organization of the adult lens and produces a clear “Organelle Free Zone” (OFZ) in the inner cortex [1]. The maintenance of the OFZ, is necessary for normal function and clarity of the adult lens [1-10]. Many things may interfere with the development of this highly organized structure of the lens that can lead to cataract formation. It is not yet known exactly which alterations lead to age related cataract (ARC) [1,10,11]. We have previously reported that an age-related failure to resolve and degrade nuclei in the lens fiber cells results in an accumulation of undegraded nuclei in the bow regions of old non mutant, untreated mice that spreads with aging to the anterior and posterior cortices [12,13] These cortical areas of age-related LEC nuclei and nuclear fragment retention also express strong reactive oxygen species (ROS), and the appearance of the ROS and extended bow region correlate to increases in opacity in the lenses of old mice [3,12,14].

**Figure 1:**
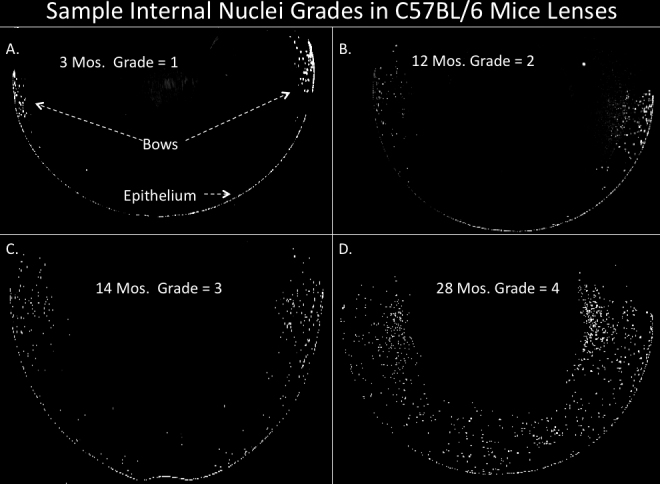
Typical examples of Internal nuclei grades using C57BL/6 lens sections from mice of different ages stained with Dapi (white). **A**: Typical staining of 3 month old mice with normal internal nuclei pattern at the bow only. **B**: At 12 months the bows become extended inwards and anteriorly (grade=2.0). **C**: by 14 months of age the Bows accumulate even more nuclei and extend extensively underneath the anterior epithelium (grade=3.0). **D**: In old age, the interior nuclei may fill in the whole anterior outer cortex (grade=4.0). Staining was done on paraffin sections from whole eyes, cut through the middle (anterior to posterior) of the lens (see Methods). The non-lens portions of the section (retina, cornea, Iris) have been filled in with black for easier comparisons. Original magnification was 40×.

**Figure 2:**
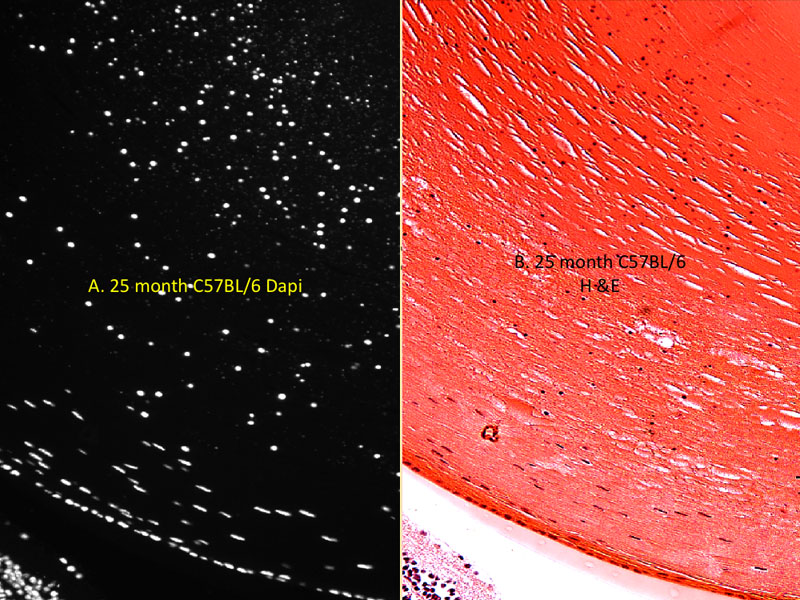
Typical nuclei and nuclear fragment cortex inclusions in a 25-month-old C57BL/6 mouse. A comparison of nuclei and nuclear cortical fragments in the bow areas of a typical 25 month-old C57BL/6 mouse eye section using both Dapi (**A**) and H&E (**B**) stains. All of the included nuclei stained with Dapi (white) were also stained blue by Hematoxylin. All other H&E and Dapi images demonstrated the same correspondence (not shown), but Dapi was much easier to score at lower magnification so was generally used in scoring and grading retained nuclei and nuclear fragments. Original magnification was 200×. Other details are as described in legend to Figure 1.

**Figure 3:**
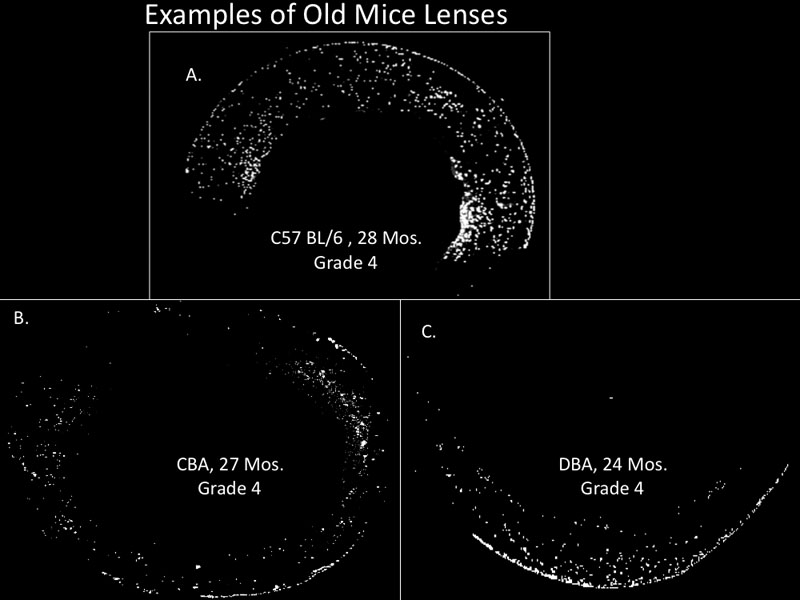
Examples of retained nuclei in three different strains of old mice. **A**: A typical C57BL/6 mouse at 28 months old. **B**: CBA at 27 months old mouse lens that was the most affected with internal nuclei. **C**: A typical old DBA mouse at 24 month. See Figure 5 for analyses of multiple mice. Other details as in legend to Figure 1.

Because the nuclear fragment retention in the lens is directly related to aging in mice and would appear to be light-obstructive [3,12-14], we wished to determine whether nuclear retention correlated with the development of age related cataracts (ARC), – appearing with advancing age in all species that develop cataracts. Cataracts or advanced lens opacities were seen by slit lamp in all of the aged mouse strains and one of the two rat strains, as previously reported [12,14]. To our knowledge, this age-related, 4'6' diamidino-2-phenylindole (DAPI) positive nuclear fragment retention in the lens cortex has not previously been described as such in species other than mice and rats [3,12-14]. However, a similar retention of nuclei or large fragments of nuclei has been reported in mutant mice [15-24] and in irradiated mice [13]. This nuclear retention first becomes noticeable in mice at midlife (12–15 months) and becomes more marked with advancing age, reaching a maximum in 28–30 month old mice and paralleling the development of lens opacity in mice (Figure 1). DAPI and hemotoxylin and eosin (H&E) nuclear stains identify the cortical fragments of nuclei and some whole nuclei as being of DNA content and their appearance and positioning strongly suggest that they are the result of a failure to resolve the nuclei of lens fiber [25]. In the advanced state that occurs with aging they extend both anteriorly and posteriorly and deep into the cortex [12-14] and see Figure 1, Figure 2, and Figure 3. Confocal examination using dihydrorhodamine uptake showed that they co-locate with ROS presence [14]. Having reported this phenomenon in one strain of mouse and one strain of rat [12,14], we wished to determine whether this phenomenon was present in all old animals of all species, where it might contribute to or even be responsible for age-related cataract. To determine if this was a generalized phenomenon we undertook to investigate three strains of mice, two strains of rats, dog, monkey and human aged lenses, with the help of the several sources listed in the acknowledgment section.

## Methods

### Animals

#### Slit Lamp examinations

The living animals (mice and rats) were examined for degree of lens opacity and the presence of a mature cataract was determined using a KOWA 15 hand held slit lamp (KOWA, Tokyo, Japan). The animals’ eyes were first dilated with a 3:1 mixture of 1% tropicamide and 10% phenylephrine hydrochloride, both for human use.

#### Determination of mouse and rat lens opacity

Living rats and mouse eyes were assessed at the indicated ages for degree of opacity by an experienced blinded viewer (N.S.W.) [3,13,26-28] with examination for stage of lens opacity rated 0 to 4 by half steps (0, 0.5, 1, 1.5 etc.). Zero was given for complete lens clarity and ability to see the retina. A rating of 3.5 or 4 was given to lenses in which there was nearly complete or complete reflection i.e., non-passage of the slit lamp beam through the lens. These same animals were then sacrificed and their eyes 4% buffered formalin fixed and sectioned. The sections were read microscopically for retained nuclei and nuclear fragments compared to young mouse or rat lenses that had only the normal minimal content at the lens bow and were therefore rated 1.

#### Dogs

Ten percent buffered formalin fixed eyes from old dogs volunteered by owners for euthanasia because of terminal status and with donor permission forms signed by their owners were sent from the Veterinary Clinics of Kansas State University, Manhattan, KS. Embedding, sectioning, Dapi, and H&E analysis done microscopically at the authors’ laboratory. Lens content of nuclei and nuclear fragments were read and rated as in the mice and rats.

#### Monkeys

*Macaca mulata* (rhesus) and *M*. *nemistrima* (pigtailed macaque) monkey eyes prefixed with 10% formalin were contributed courtesy of Dr. Julie Mattison of the National Institutes on Aging primate colony, NIH, Baltimore, MD, and by Dr. Martha Neuringer, Department of Neurology and the University of Oregon Primate Center, OHSC, Portland, OR. These were fixed at the above sites immediately after the death of animals used for other research purposes or dying of natural causes. Embedding sections and readings were made at the authors’ laboratory similar to the dogs.

#### Humans

Buffered formalin fixed human lenses were obtained from both National Disease Research Exchange (NDRI; Philadelphia, PA) a national human tissue distribution center, and from the Lions Eye Bank (later named Sight Life) located in Seattle, WA. These organizations each had their own oversight committees and donor or donor family permission programs that allowed them to furnish tissue samples to the many recipients of the tissue for a price. Thus it was not necessary for the present authors to institute their own permission program or that of the University of Washington. Embedding, sections and readings were made at the authors’ laboratory similar to dogs.

#### Total individuals of each species used in experiments

For C57bl/6 mice only separate cohorts were used for determination of retained nuclei, cohort #1, or opacity, cohort #2. Mice used: C57BL/6 used for nuclei staining (cohort #1): at 3 months of age 8 eyes from 8 donors, at 5 months 5 from 5 donors, at 12 months 4 eyes from 4 donors, at 14 months 4 eyes from 4 donors, at 28 months 8 eyes from 8 donors. C57BL/6 used for opacity measurements (cohort 2): at 8 months 16 eyes from 8 donors, at 16 months 16 eyes from 8 donors, at 24 months16 eyes from 8 donors, at 32 months 8 eyes, from 8 donors were used. CBA mice: at 27 months, 8 eyes from 4 donors were used. DBA/2 mice at 24 months: 8 eyes from 4 donors were used. Brown Norway rats at 4 months 8 eyes from 4 donors, at 31 months 8 eyes from 4 donors BNF1 rats at 4 months 4 eyes from 4 donors, at 16 months 4 eyes from 4 donors, at 31 months 4 eyes from 4 donors. Dogs: Ten eyes from 7 old donors were used at 9–17 years of age. Monkeys: 22 eyes from 20 donors at 2.5 to 38 years old. Humans: 10 eyes from 7 old humans at 64–88 years of age. Some individual figures also indicate when the lenses shown are “typical” and thus where only one of each type is shown for comparison. The same animals in the CBA, and DBA mice and the 2 strains of rats were used for both lens opacity and nuclear inclusion measurements. Of necessity, the dogs, monkeys, and human eyes were examined only for nuclear inclusions.

### Tissue handling and lens classification

The old dog, monkey, and human eyes or lenses were fixed at the sender’s site before shipment and thus slit lamp examinations were not performed in most cases. Mouse and rat eyes were fixed on our site after slit lamp examination of the live donors. The dog donors’ eyes were taken at time of euthanasia, the monkey eyes at time of various terminal experiments or immediately after age-related death, and human eyes within 6 h of natural death and fixation in buffered 10% formalin. Dog and monkey eyes were obtained at 70%–90% of estimated life span, and humans only from old donors, as noted in Appendix 1. The mouse and rat eyes were taken at time of sacrifice at the times indicated below. For life spans of each species see- dogs [29], monkeys [30], humans [31-33], for mice and rat strains [26]; and also Jackson Laboratories strain lifespan data for mice [34].

### Immunohistochemistry

Whole eyes were removed placed in DMEM with Hepes. The eyes were opened slightly at the optic nerve to promote entry of the fixative. Then the whole eye was placed in 3.7% paraformaldehyde, pH 7.4 plus 4% sucrose for one hour at room temperature. After fixation, the eyes were washed in PBS, then processed into paraffin blocks and sectioned at 10 microns onto slides. Subsequently, slides were deparaffinized, then mounted and stained with hematoxylin and eosin (H&E) or Vectashield with dapi, (Vector Laboratories Inc., Burlingame, CA). The microscope used was a Nikon Eclipse E600 with a QImaging Retigia EX CCD Camera (Melville, NY). The basis for histological lens status classification was the degree of the cortex inclusion of nuclei or nuclear fragments. A rating of 1 to 4 was given for number and spread of retained nuclei and nuclear fragments in the lens cortex (1.0 being reserved for the lens of a young animal with a normal sub-equatorial lens fiber cell status).

### DLAD immunofluorescence by biotin-streptavidin staining

The antibody to native mouse DLAD was given to us by Dr. S. Nagata (Kyoto University, Kyoto, Japan) [35]. Deparafinized sections were blocked for 1 h. with 10% BSA (BSA) dissolved in PBS then further blocked with streptavidin-biotin blocking kit (00–4303; Invitrogen, Camarillo, CA) to reduce background stained. Primary Armenian hamster DLAD antibody (1.5 mg/ml) was diluted 1:100 with 3% BSA in PBS was applied to the sections overnight at 4 °C. After washing, a secondary biotynilated anti-Armenian hamster antibody (Vector Laboratories Inc.) was diluted 1:200 and added for 90 min at room temperature in 3% BSA, PBS buffer. The sections were washed exhaustively with PBS and then incubated with a 1:200 dilution of Streptavidin conjugated to Alexa Fluor® 488 (Cat. No. S-32354; Invitrogen, Camarillo, CA) in 3% BSA, PBS. Following final washing the sections were post-fixed with 3% paraformaldehyde in PBS, and mounted with Vectashield with DAPI to stain DNA (Vector Laboratories Inc.).

### PCR assay of DLAD mRNA

Mice were killed by cervical dislocation. Lenses were immersed in RNAlater (Qiagen) and frozen at −20 °C for future use. Due to their small size two lenses from the same 3-month-old animal were pooled for subsequent RNA isolation. The large size of lenses from 27-month-old animals allowed each lens to be processed independently. Lenses were ground up in the presence of RNAlater with a micro pestle, then additional RLT buffer containing beta-mercaptoethanol from the RNAeasy micro kit was added following the protocol from Qiagen. MRNA was extracted from cell lysate using the Dynabeads mRNA direct kit following the protocol from Invitrogen. cDNA was produced using SuperScript II reverse transcriptase following the protocol from Invitrogen. After first-strand synthesis, purity and concentration of the cDNA was measured with a spectrophotometer. PCR primers from TaqMan and FastStart Universal Probe master mix from Roche (Pleasanton, CA) were combined with cDNA in rotorgene tubes from Mt Baker Bio (Everett, WA). Quantitative real time PCR was performed using Taqman primers following the protocol from Applied Biosystems (Carlsbad,CA).

Triplicates of each reaction were run in the Rotor Gene 6 apparatus (Qiagen). The housekeeping gene glyceraldehyde-3-phosphate-dehydrogenase (*Gapdh*) was used as an internal reference. Universal mouse cDNA (BioChain; Hayward, CA) was used as an external reference. Negative controls containing no cDNA were present in every PCR run. Taqman primers (Applied Biosystems) used were: *Gapdh* Mm99999915_g1 and Mm03302249_g1; Dnase2b (*DLAD*) Mm004098304_m1; X box binding protein (*Xbp1*) Mm00457357_m1; heat shock protein 90 (*Hsp90b1*; *Grp94*) Mm00441926_m1; heat shock protein 5 (*Hspa5*; *BiP*) Mm00517691_m1; DNA damage-inducible transcript 3 (*Ddit3*; *CHOP*) Mm00492097_m1; pancreatic lipase related protein 2 (*Pnliprp2*) Mm00448214_m1; and lysosomal associated membrane protein 1 (*Lamp 1*) Mm00495262_m1.

### Statistical analysis

Analysis was performed using PROC UNIVARIATE, PROC NPAR1WAY and PROC GLM in the SAS System ® version 8.02. Regression coefficients were determined in Microsoft Excel®. For within-species analysis, ages were considered the dependent variable. For comparison between-species analysis, the oldest individual for every species was considered to be 100%, all other ages were set as a percentage of this maximum age.

## Results

**Figure 4:**
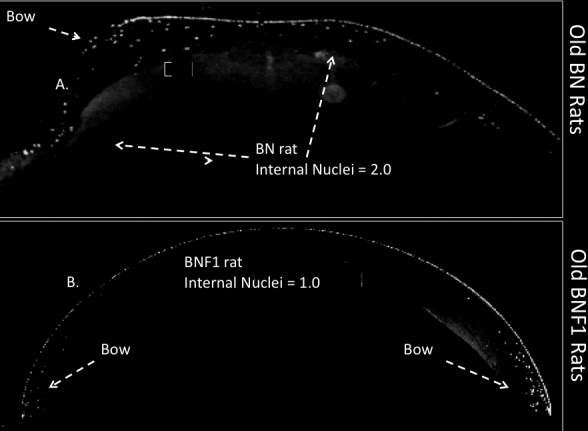
Internalized nuclei in 31-month-old BN and BNF1 rats. Examples of **A**: old Brown Norway (BN) and **B**: old BNF1 rat lenses sections stained with Dapi to show typical internal nuclei. Both rats were 31 months old when sacrificed. The young BN and BNF1 rats (not shown) appeared just like young mice with no included nuclei, and discreet bows. Other details as in the legend to Figure 1.

**Figure 5:**
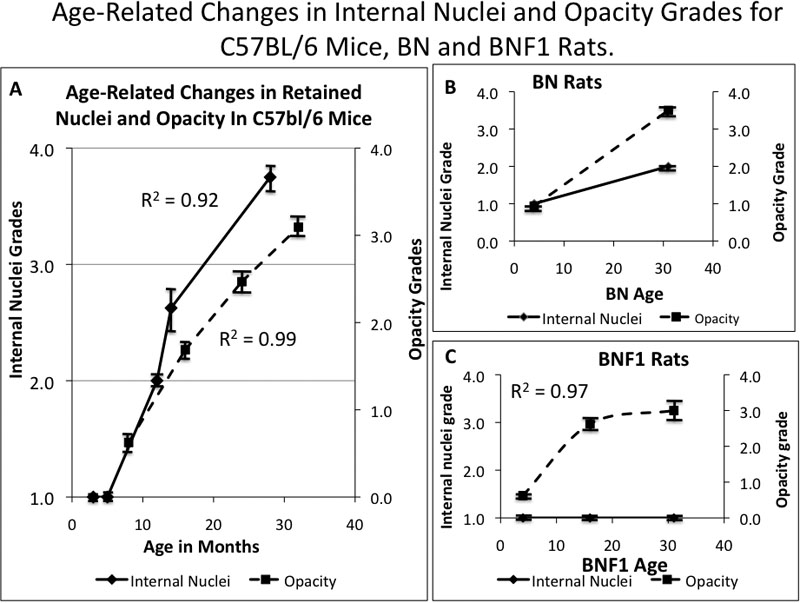
Age-related progressive change in internal nuclei grade and slit lamp opacity grade in C57BL/6 mice and 2 strains of rats. **A**: For C57BL/6mice the opacity grades and retained nuclei grades were determined using two separate cohorts of C57BL/6 mice, see Appendix 1. C57bl/6 mice eyes used for nuclei staining (cohort #1) included 8 eyes from 8 donors at 3 months of age; 5 eyes from 5 donors at 5 months; 4 eyes from 4 donors at 12 months; 4 eyes from 4 mice at 14 months, 9 eyes from 9 donors at 28 months. For C57BL/6 opacity measurements (cohort #2): 16 eyes from 8 donors at 8 months, 16 eyes from 8 donors at16 months; at 16 eyes from 8 donors at 24 months, ; 8 eyes, from 8 donors at 32 months. **B**: Age-related changes in BN rats determined for 8 eyes from 4×4 months old rats and 8 eyes from 4×31 month old rats. **C**: Age changes for BNF1 rats determined for 4 eyes from 4×4 months old, 4 eyes from 4×14 month-old rats, and 4 eyes from 4 mice at 31 months-old. Note that in the BNF1 the internal nuclei do not increase with age nor correlate to opacity grade, but in Brown Norway they do. Lines are fit by eye, and the regressions (R2) are for a logarithmic fit to the data unless otherwise stated. Error bars represent standard errors of the means. Other details are as described in legend to Figure 1.

Figure 1 shows typical nuclei and nuclear fragment retention in the cortices of C57BL/6 mice of several ages stained DAPI . Nuclear retention is rated from 1, a normal equatorial bow, to a maximum of 4. The retained nuclei and fragments first build up at the bow then spread anteriorly and posteriorly eventually filling the whole outer cortex in all old C57BL/6 mice. The retained fragments can also be seen with H&E staining which shows the same pattern of retained nuclear material (Figure 2). In Figure 3 we compare DAPI stained sections from typical old C57BL/6 with two other strains of mice: old DBA/2 and the most affected old CBA mouse. Extreme expansion of retained nuclei is seen in all of the DBA/2 mice, but only one of the old CBA mice. Figure 4 shows typical sections from 31 month-old BN and BNF1 rats. Only the BN rat demonstrated a mild age-related increase (see also Figure 5). Figure 5 shows age-related increases in retained nuclei and opacity (slit lamp grades) in C57BL/6 mice and in both strains of rats. Old BNF1 rats do not retain nuclei at all (grade 1) in, but old BN rat do to some extent (average grade=2.0) although not as extensively as the mice (Figure 5, Appendix 1, Appendix 2, and Appendix 3). Again opacity is always closely correlated with age in the C57BL/6 and DBA/2 mice, but less so in CBA mice and BN rats and not at all in BNF1 rats. The status of young (no inclusions) and old BN rats is given in Pendergrass et al. [12].

**Figure 6:**
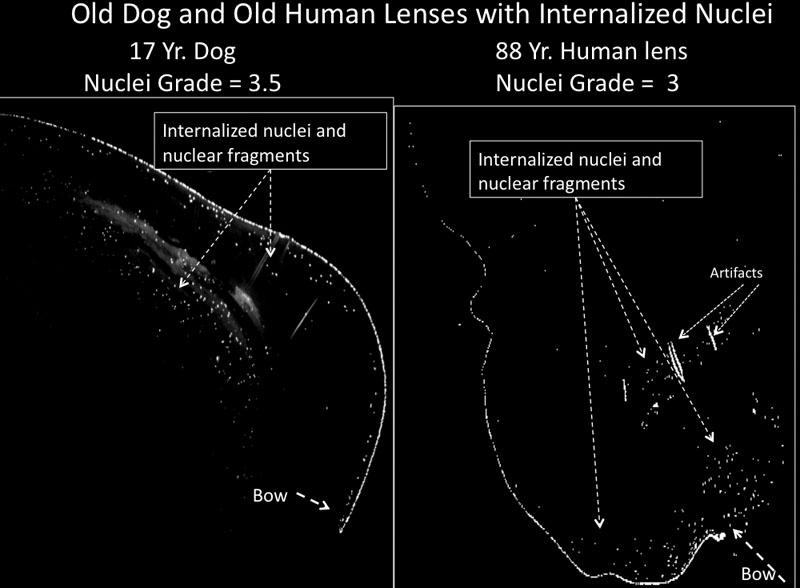
Examples of old dog and human lenses with nuclei retention. **A**: 17-year-old affected dog lens showing retained nuclei. **B**: An 88-year-old human lens with retained nuclei. Some dog and human lenses were not affected. Other details are as described in legend to Figure 1.

**Figure 7:**
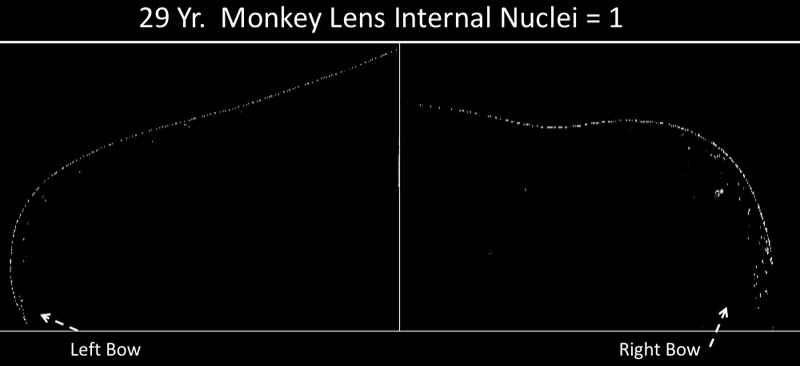
Left and right halves of a 29 year typical old Rhesus monkey lens stained with Dapi to show lack of internalized nuclei. None of the old monkeys demonstrated any significant increase with age in internal nuclei or nuclear fragments although two were noted to have cataracts by the veterinarian at the site of origin. Other details are as described in legend to Figure 1.

**Figure 8:**
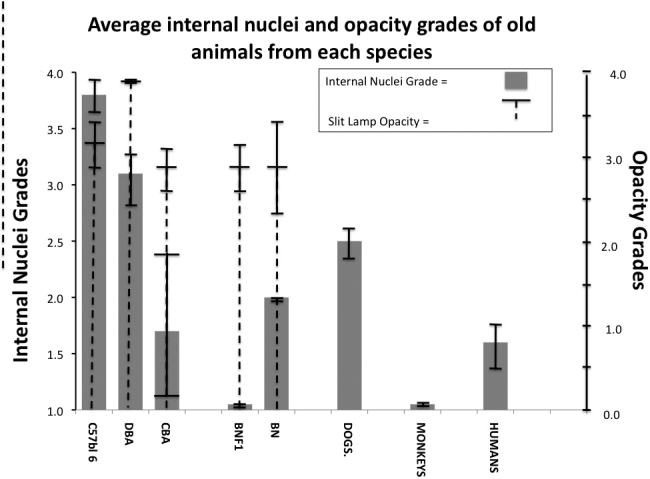
Comparisons of internal nuclei and fragment retention (gray columns) and opacity by slit lamp (dashed lines). The grades are for old individuals (80%–100% of maximum lifespan) from 8 strains from 5 species. C57BL/6 mice for nuclear retention grades: 9 eyes from 9×28 month-old mice; for opacity the average of 8 eyes (from 8 mice) at 24 months and 8 eyes (from8 mice) at 32 months. For the DBA mice: 8 eyes from 4×24 month old DBA mice/ For CBA mice: 7 eyes from 4×27 month-old CBA mice. Rats: 4 eyes from 4×31 month-old BNF1 rats, 8 eyes from 4×31 month-old Brown Norway rats (BN). Dogs: 10 eyes from 7 old dogs from 9 to 17 years, mean age=12 years, opacity in dogs not regularly noted at supplier site, although one noted to have cataract. Monkeys:16 eyes from 15 old female and male rhesus (19–38 years), mean=24 years opacity not regularly checked, but 2 individuals noted to have cataracts. Humans: 10 eyes from 7 elderly male and female human autopsies (64–88 years, mean=80 years). Opacity in humans was not regularly checked, but one individual was noted to have bilateral cataracts. Other details are as in the legend to Figure 1. For species life spans see Methods references.

**Figure 9:**
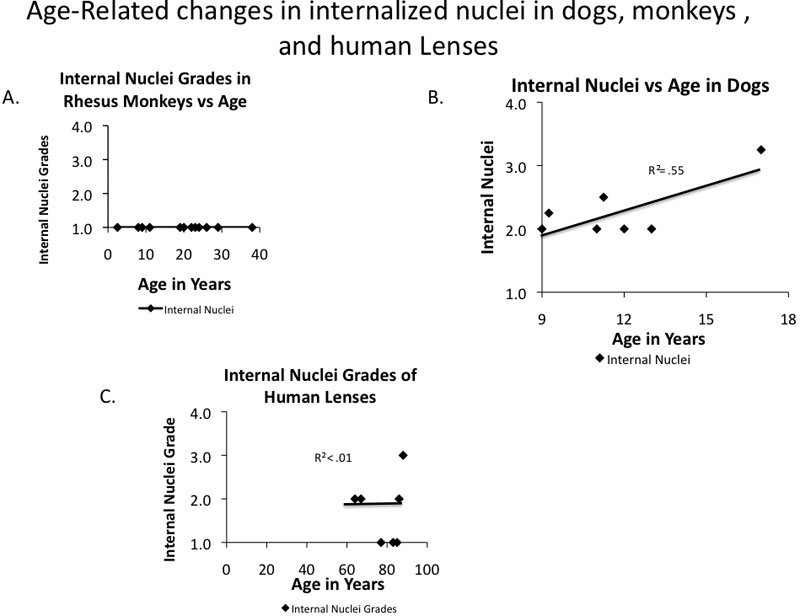
Internalized nuclei in monkeys, dogs, and humans. Internal nuclei and nuclear fragment grades for individual **A**: Rhesus Monkeys, 20 individual monkeys; **B**: Dogs, 7 individual dogs; and **C**: Humans, 7 individuals. Only relatively old dogs and human lenses were available. Each point represents average values for one animal. Other details are as described in legend to Figure 1.

We also examined dog, human (sample sections in Figure 6), and monkey lenses (Figure 7) for retained nuclei. A summary comparing the averages of the old animals from each species is shown in Figure 8. Because the eyes for these three species were fixed at distant laboratories we could not provide slit lamp readings for the dog, monkey and human lenses, so only data for nuclear retention ratings are given (Figure 8, Figure 9, and Appendix 4). However, at least two monkeys, one human, and two dogs were noted to have obvious cataracts by the veterinarian at the distant site. Old dog lenses did have increased nuclear retention with an average grade of 2.5, but monkeys had no individuals with grades above 1 (Figure 8 and Appendix 4). One of the humans (the oldest at 88 years) had a score of 3.0 but the average for all old humans was only 1.6 indicating serious retention of nuclei was only sporadic in old human lenses.

In summary the retention of nuclear fragments with animal age is universal in 2 strains of mice (C57 and DBA/2) less extensive in CBA mice and in BN rats, in dogs and sporadically in humans, while not al all present in old monkeys or in old BNF1 rats despite quite marked increases in the opacity readings for all mice and rats (see Figure 8, Appendix 1, Appendix 2, Appendix 3, and Appendix 4).

### Statistical analysis of nuclear retention and opacity

In C57Bl6 mice (Figure 5 and Appendix 1) we observed a highly significant influence of age on the presence of retained nuclei in the lens (p<0.0001), as well as a highly significant influence of age on opacity (p<0.0001). When merging the DBA and CBA mice into the data (Appendix 2), this influence remained significant for both the degree of internal nuclei and for opacity (p=0.0002 in both cases).

In rats (Figure 5 and Appendix 3), age had a significant influence on nuclei overall (p<0.0001); however, this effect was present in BN rats only. Age also had a significant effect on opacity (p<0.0001). When limiting the analysis to BNF1 rats, opacity remained significant (p=0.0008), but no significant influences of nuclei were found (p=1), while in BN rats, the presence of nuclei and opacity were highly correlated (p<0.0001, R^2^=0.939). In both strains, the generalized linear model was highly significant (p<0.0001, R^2^=0.865). Opacity (p=0.001) was a highly significant predictor of age; the interaction of opacity and nuclei was not quite significant (p=0.052), and nuclei were also significantly affected by age (p=0.031).

In dogs (Figure 5 and Appendix 4), opacity values were not available and were, therefore, not considered in the calculations. There was a significant influence of age on nuclei (p=0.038). The minimum age of any dog in the data was 9 years, which means that there were no young dogs, which means that this result would likely be significant if younger dogs were considered (also see our dog cataract paper [36]). The number of dogs was just 7, which also limits the statistical power of this analysis.

In monkeys (Figure 9 and Appendix 4), opacity was a yes/no measurement and therefore taken as the class variable. No nuclei were present in any of the monkeys so internal nuclei grades were not correlated with age at all. Age was not a significant predictor of opacity (p=0.455); however, there were only two positive cases out of 22 exams.

In humans (Figure 9 and Appendix 4), opacity was also a yes/no measurement, but nuclei were present in some individuals. Ten samples could be analyzed overall. Age was not a significant predictor of opacity as only one human lens was classified as cataractous when received (p=0.346). Age was also not a significant predictor of nuclei (p=0.287). In the generalized linear model, neither the model (p=0.910), nor nuclei (p=0.978) were significant, while opacity and its interaction with nuclei did not yield useful p-values. This can probably be explained by the low number of samples (n=10) and the absence of young individuals.

### Quantitative PCR analysis of mRNA (qPCR)

The relative levels of *DLAD* mRNA in old and young lenses from C57BL/6 mice were determined using qPCR and the results are shown in Table 1. DLAD is a lysosomal enzyme essential for degradation of lens DNA in differentiating lens fibers [25,37,38]. Two other lysozomal enzymes, Pnliprp2, and Lamp 1, a universal marker for lysozomes were also analyzed by qPCR, as were several proteins involved in the Unfolded Protein Stress Response including, XBP1, a transcription factor that activates unfolded protein response genes, HSP90B1, a molecular chaperone that plays a role in folding newly synthesized proteins that have been denatured, HSPA5 (BiP or Grp78), a protein involved in folding and assembly of proteins in the ER, and DDIT3 (CHOP) a protein activated by ER stress which promotes apoptosis. Of these only DLAD decreased significantly with age. The mRNA values of the others were the same from old and young lenses (not shown).

**Table 1:** The extent of loss of DNase II-like acid DNase-beta (DLAD), during aging of C57BL/6 mice.

**Age**	**(Average C_T_*DLAD* – average C_T_*Gapdh*)**	**2-C_T_*DLAD* – C_T_*Gapdh***	**Mean±SEM**	**Ratio of old/young**
27 months old	8.3	0.0032	0.0036±0.0010	13.1%
	7.6	0.0052		
	8.7	0.0024		
3 months old	6.0	0.016	0.0272±0.005	13.1%
	5.0	0.031		
	5.4	0.024		
	4.7	0.038		

The mRNA concentration is defined as the PCR threshold cycle time (CT; time when the cDNA fluorescence reaches 1.1× of the baseline fluorescence) as a power of 2. Each individual cDNA sample was normalized to the concentration of the control mRNA, Gapdh. The average CT for *Gapdh* (control enzyme) was subtracted from the average CT for DLAD (column 2). Because PCR amplification is exponential, the difference in CT s must be converted by a power function (column 3). This relative ratio of [mRNA *DLAD*]/[mRNA *Gapdh*] is given by the Equation 2exp -(CT *DLAD* -CT *Gapdh*). Comparing the values from four 27 month old lenses to the values from three 3 month old lenses, we find a p value <0.01 indicating that mRNA for *DLAD* was significantly reduced in the old lenses. SE=standard errors of the mean. The analyses were performed in triplicate on lenses from 3×c57bl/6 young mice at 3 months of age compared to lenses from 4 old mice at 28 months.

Thus 27 month old mouse lens had only 13% of the *DLAD* mRNA found in 3-month-old mouse lenses (p<0.01), but none of the other proteins decreased significantly with age in C57BL/6mice, the most heavily affected animals. Lack of DLAD may directly contribute to the retention of nuclei and nuclear fragments in mouse lenses [1,35,37,38].

**Figure 10:**
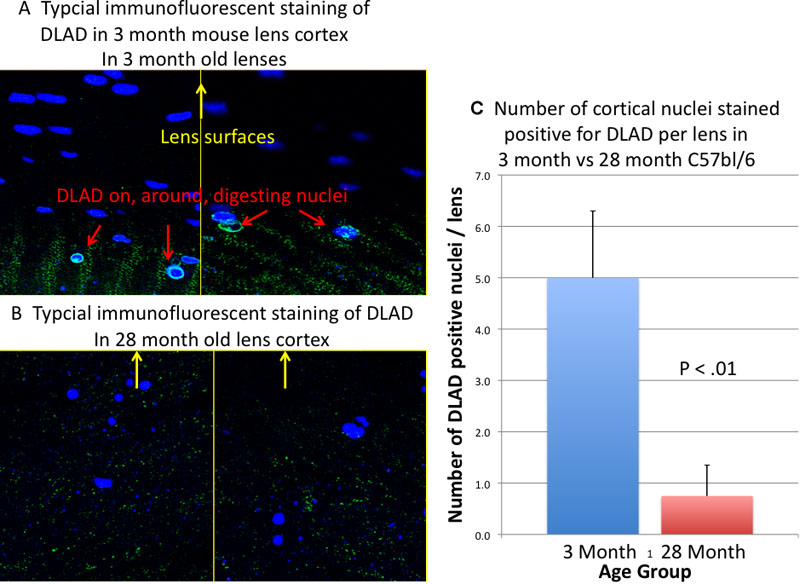
DLAD staining and imaging was done on eye sections with Nagata antibody to native DLAD as described in the Methods. **A**: Typical DLAD immunofluorescence (Green, red arrows) of cortical nuclei (blue) from 2 different 3 month old C57BL/6 mice lenses, 630× original magnification. Green DLAD circles were only seen in cortical nuclei at the deepest levels before digestion. **B**: Similar staining of 28 month old C57BL/6 mice lenses. Note lack of green DLAD circles on blue nuclei. Yellow arrows show direction of lens surface. **C**: Comparison of the total number or cortical nuclei (per lens) stained with DLAD antibody in 3 month vs 28 month C57BL/6 mice lenses. One lens from each of 4 different c57BL/6 mice at 3 months and at 28 months were analyzed for comparison. The green stippling is background staining. Error bars represent the standard errors of the means. The p value is for a 1-tailed Ttest comparing four animals of each age.

We also examined staining of DLAD protein using an antibody to the native mouse enzyme from Dr. S. Nagata [38] by streptavidin-biotyn immunofluorescence. These results shown in Figure 10 are consistent with the qPCR data. The DLAD antibody as seen in Figure 10 formed rings around degenerating nuclei at the base of the bow. The 3 month old c57bl/6 sections had an average of 5 such DLAD rings per lens, and the 28 month mouse lenses had only 0.8 rings per eye=16% of the young lenses (p<0.01).

## Discussion

Our overall findings are that there is an age-related increased retention of nuclei and nuclear DNA-containing fragments in the cortex of old mice, and these are present far beyond the cortical bow region, whereas in younger mice through early middle age there is a degradation of these nuclei from the maturing lens fiber cells shortly after lens surface cells enter the cortex at the equator. We emphasize that this is not of moderate degree but of greatly extended nuclei and nuclear fragment presence beyond the normal bow region [1,32,39,40]. As indicated by DAPI and H&E, these are nuclei and nuclear fragments containing DNA from the lens fiber cells. While these may be coincident in time with the well established protein changes that lead to cataract, they clearly represent a failure to dissolve or otherwise remove the nuclei of the lens fiber cells [12-14,25,35,38]. Unlike some mutant mice, there was no diminution of eye size or early life, changes in lens fiber cells or lens fibers in the young normal animals. Rather, beginning at ~12 months of age there was a reduction of the removal of nuclei and nuclear remnants in the maturing lens fiber cells in the extended cellular bow of the lens cortex, and this accretion extended in depth and distance anteriorly and posteriorly throughout the lens with advancing mouse age. As discussed below, we find this phenomenon to exist universally in C57BL/6 and DBA/2 mice with partial presence in CBA mice, BN rats, and mid-sized old dogs, but only sporadically in humans and not in any monkeys or BNF1rats. We found, as we had previously, that lens opacity increased with age in all of the animals in which it was followed and eventuated in mature cataract in many of the old animals [13,14,26,27].

There have been many studies and reviews of the processes and the mechanisms involved in the changes in lens fiber cells to preserve the organelle free zone (OFZ) of the lens cortex [1,10,41-43]. The factors present in the old animal cortices and listed as refracting or scattering light rays, and therefore contributing to ARC development, and include laminar inclusions and changes in lens fibers and lens fiber cells, as noted in reviews [1,43]. Given this, it is of interest that the retained nuclei and nuclear fragments reported by us are DAPI positive and always appear in the old mouse cortex but do not always accompany the development of lens opacity or mature cataract in all old animals of other species. This is obvious in our slit lamp and microscopic section correlated viewing. It is noteworthy that the nuclei and nuclear fragments were not present in the lenses of one strain of old rat, BNF1, and in any old monkey lenses at a very late stage of life, a period when they are very prominent in mouse lenses.

We note that the lens opacity increases with age in living old monkeys, as previously noted by slit lamp examination by one of us (NSW, not shown). Thus, we can say that the nuclei and nuclear fragment retention does not appear in old monkeys, regardless of lens opacity. Both slit lamp determined opacity and histological nuclei were found in Brown Norway rats but only slit lamp opacity and not nuclei retention was found in the hybrid BNF1 rats of similar old age.

The presence of nuclear fragments has been remarked upon in mutant and/ or stressed mouse models by other experimenters, where they often appear early in life [1,16,17,19,22-24], while we have previously reported their universal presence in old non mutant mice and BN rats and at an early time in irradiated mice [13].

The mechanism responsible for organelle degradation in the lens cortex is not completely known [1]. However, the lysozomal enzyme DLAD has been shown to play a key role in degradation of lens nuclei during normal development [1] and cortical lens nuclei are not degraded in recombinant mice lacking the enzyme [35,37,38]. In preliminary experiments to determine the cause of nuclear retention in old animals we examined this key enzyme. In old mice lenses, DLAD message was reduced to 13% of young controls. This alone could account for the lack of nuclear degradation in the old but non mutant mice cortices [35,37,38]. We examined message for two other lysozomal enzymes (see Results) but they were unchanged in old lenses, as were several enzymes involved in the unfolded protein stress response (See Results). As shown in Figure 10, there was also less staining of the native DLAD protein in lenses from old cataractous C57BL/6 mice. We were unable to procure fresh material from other species to examine in each species DLAD loss with age. The reason for the heterogeneity in DLAD loss with aging and cataract appearance in different species is not known, but a related finding has been reported by Nagai et al. [37] who reported that one type of early onset heritable cataract in rats (the UPLR rat) correlates with loss of DLAD and build up of undigested DNA, but in another cataractous model, (the SCR rat), cataracts occur without DLAD loss or retention of cortical DNA. Thus it is possible for loss of DLAD to be a factor in age-related cataract appearance some strains and species and not in others.

In conclusion, we have demonstrated that age related slit lamp viewed lens opacity and age related nuclei/nuclear fragment retention do not correlate in several species and one rat strain. The retention of lens fiber cell nuclei and nuclear fragments do not appear at all by microscopic examination in the lenses of old monkeys and one strain of old rats, with the latter shown to have advanced lens opacities in the same animals then microscopically studied for retained nuclei. We note that the lenses of all species examined were obtained at an advanced age in which they express advanced lens opacity. Our conclusion must be that the two findings on lens status do not agree in some species and strains. Our present findings do indicate the incomplete removal of fiber cell nuclei in the process of maturation to lens fibers in the aging mouse coincides with a loss of DLAD mRNA [25,35,38]. However this event (loss of DLAD) likely does not extend to all other species where normal degradation of fiber cell nuclei occurs in old animals, such as the monkey or BNF1 rat, and it cannot contribute to lens opacity there. This should be taken into consideration especially when using the mouse as a model for age-related cataract in humans and other primates.

## Appendix

Appendix 1. C57BL/6 mice internal nuclei grades, and opacity. The internal nuclei grades were scored on a separate cohort of mice (**A**) than the opacity grades (**B**). Opacity was determined by slit lamp (see Methods). Other details as in the legend to Figure 1. In the opacity readings in **B**, the opacities are averaged for right and left eyes from each animal. To access the data, click or select the words “Appendix 1.” This will initiate the download of a compressed (pdf) archive that contains the file.

Appendix 2. Individual internal nuclei and opacity grades for DBA/2 and CBA old mice. Other details as in legend to Figure 1. Individual internal nuclei and opacity grades for DBA/2 and CBA old mice. To access the data, click or select the words “Appendix 2.” This will initiate the download of a compressed (pdf) archive that contains the file.

Appendix 3. Individual internal nuclei and opacity grades for BN and BNF1 rats. Other details as in legend to Figure 1. To access the data, click or select the words “Appendix 3.” This will initiate the download of a compressed (pdf) archive that contains the file.

Appendix 4. Individual internal nuclei and opacity grades for dogs (**A**), Rhesus and Pig Tail monkeys (**B**), and humans (**C**). Other details as in legend to Figure 1. To access the data, click or select the words “Appendix 4.” This will initiate the download of a compressed (pdf) archive that contains the file.
